# Supplementary material for: Sensor data to measure Hawthorne effects in cookstove evaluation
Source: Data Brief. 2018 Apr 11;18:1334–9. doi: 10.1016/j.dib.2018.04.021 (PMC5997008; doi:10.1016/j.dib.2018.04.021)
Supplement: Supplementary file 2 — Supplementary material [file mmc2.zip › Data_in_Brief/2017-05-10 Data in Brief Simons.docx]

*Data article*

**Title: *Sensor data to measure Hawthorne effects in cookstove evaluation***

**Authors:** Andrew M. Simons, Theresa Beltramo, Garrick Blalock, David I. Levine

**Affiliations:** Simons: Department of Economics, Fordham University (email: asimons5@fordham.edu). Blalock: Charles H. Dyson School of Applied Economics and Management, Cornell University (email: garrick.blalock@cornell.edu). Beltramo: United Nations High Commissioner for Refugees, Geneva (email: [beltramo@unhcr.org](mailto:beltramo@unhcr.org)). Levine: Haas School of Business, University of California, Berkeley (email: [levine@haas.berkeley.edu](mailto:levine@haas.berkeley.edu)).

**Contact email: asimons5@fordham.edu**

**Abstract**

*This data in brief article includes estimated time cooking based on temperature sensor data taken every 30 minutes from three stone fires and introduced fuel-efficient Envirofit stoves in approximately 168 households in rural Uganda. These households were part of an impact evaluation study spanning about six months to understand the effects of fuel-efficient cookstoves on fuel use and pollution. Daily particulate matter (pollution) and fuelwood use data are also included. This data in brief file only includes the weeks prior to, during, and after an in-person measurement team visited each home. The data is used to analyze whether households change cooking patterns when in-person measurement teams are present versus when only the temperature sensor is in the home.*

**Specifications Table** *[please fill in right-hand column of the table below]*

| Subject area | *Economics* |
| --- | --- |
| More specific subject area | *Environmental Economics, Development Economics* |
| Type of data | *Stata data files and do-files* |
| How data was acquired | *Temperature sensor data was gathered using SUMs (stove use monitors), air pollution data was gathering using UCB Particulate and Temperature Sensors (UCB-PATS) to measure particulate matter PM2.5 concentrations, and the kilograms of firewood used daily were gathered with a scale.* |
| Data format | *Estimates of daily minutes cooked per household stove, daily PM2.5 concentrations, daily kilograms of firewood consumed* |
| Experimental factors | *Data is cleaned and processed to include daily minutes cooked per household stove, daily PM2.5 concentrations, daily kilograms of firewood consumed* |
| Experimental features | *Cookstove usage data, fuelwood usage data, and pollution data from both three stone fire and Envirofit stove from 168 households that purchased a fuel-efficient cookstove.* |
| Data source location | *Mbarara district, Uganda* |
| Data accessibility | *Data is with the article* |

**Value of the data**

- **Data could be compared to other field studies where unobtrusive sensor data (such as cell phone monitoring, redemption of coupons, motion sensors, etc.) was gathered to see if magnitudes of Hawthorne effects are similar in different field evaluation contexts.**
- **Data provides measures of fuel-use, pollution, and time spent cooking for a field evaluation of fuel-efficient stoves and could be used to compare with other cookstove projects.**
- **Data highlights the value of gathering multiple types of data to measure outcomes for the same behavior (in this case cooking) and as such could be compared with other studies that use multiple data collection processes to refine measurement techniques.**

**Data**

We tracked stove usage before and after the purchase of a fuel-efficient stove at 168 households spread across fourteen rural parishes in Mbarara, Uganda. Upon arriving in a new parish, staff displayed the fuel-efficient stove (Envirofit G-3300) and offered it for sale to anyone who wanted to purchase at 40,000 Ugandan Shillings (approximately USD $16, see Beltramo, Levine, et al. (2015), Beltramo, Blalock, et al. (2015), and Levine et al. (2016) for additional details on the experimental setting). Consumers who wanted to buy the stove were randomly assigned into two groups (early buyers, late buyers). The project asked both early buyers and late buyers if they would agree to have stove use monitors (SUMs) placed on their traditional three stone fires immediately. Then approximately two weeks later the early buyers group received their first Envirofit stove, and approximately four to five weeks after that the late buyers received their first Envirofit stove. We also performed standard kitchen performance tests (KPTs), which include weighing the firewood pile daily to gauge fuelwood consumption and measuring indoor particulate matter concentrations with UCB particle and temperature sensors (UCB-PATS).

**Experimental Design, Materials and Methods**

Eligible households who wanted to buy the stove were randomly assigned to two groups: early buyers, late buyers. We asked both early buyers and late buyers if they would agree to have stove use monitors (SUMs) that read stove temperatures placed on their traditional and Envirofit stoves. After giving consent, three stone fires were fitted with SUMs immediately and we collected a baseline round of data with only three stone fires present in homes.

Approximately two to three weeks after the baseline data collection the early buyers group received their first Envirofit stove and we did a midline round of data collection. Then approximately five to six weeks after early buyer received their first Envirofit, the late buyers received their first Envirofit stove. About six weeks after late buyers received their Envirofits, both groups were surprised with a second Envirofit stove. Because common cooking practices in the area require two simultaneous cooking pots (for example rice and beans, or *matooke* and some type of sauce), and the Envirofit is sized for one cooking pot, we gave a second Envirofit to permit normal cooking using only fuel-efficient stoves. We then collected our endline data.

We tracked stove temperatures for approximately six months (April–September 2012). To track usage, we used small, inexpensive and unobtrusive sensors: stove use monitors (SUMs) that record stove temperatures without the need for an observer to be present.^[[1]](#footnote-1)^ Using SUMs to log stove temperatures was initially suggested by (Ruiz-Mercado et al. 2008) and has been used successfully in various settings (Mukhopadhyay et al. 2012; Ruiz-Mercado et al. 2013; Pillarisetti et al. 2014). We installed SUMs on two Envirofits and two three-stone fires (by the end of the study numerous SUMs had been lost or burned up; therefore, at the end line we measured both Envirofits and the primary three stone fire).

We also performed standard kitchen performance tests (KPT) (Bailis, Smith, and Edwards 2007a) in each household to measure the quantity of fuel wood used, record detailed food diaries, and measure household air pollution^[[2]](#footnote-2)^ at baseline, midline and endline. The KPT lasts approximately a week and involves daily visits by a small team of researchers weighing wood, monitoring household air particulate monitors, and collecting survey data on stove usage over the last 24 hours and related topics.

Throughout the study, field staff recorded about 2,400 visual observations of whether a stove was in use (on/off) when they visited homes to exchange stove usage monitors or gather data for the KPT. Then we used a logistic regression model to examine the temperature data immediately before and after the 2,400 visual observations of use. The algorithm analyzed the data to understand how temperature patterns change at times of observed stove use and then predicted cooking behaviors to the wider dataset of 1.7 million temperature readings. This process, detailed in Simons et al. (2014), allowed us to unobtrusively and inexpensively track daily stove usage on a large sample of households for six continuous months.^[[3]](#footnote-3)^

*Placement of SUMs*

SUMs must be placed close enough to the heat source to capture changes in temperatures, but not so close that they exceed 85°C, the maximum temperature the SUMs used in this study can record before they overheat and malfunction. We do not need to recover the exact temperature of the hottest part of the fire to learn about cooking behaviors. Even with SUMs that are reading temperatures 20-30 cm from the center of the fire, as long as the temperature readings for times when stoves are in use are largely different than times when stoves are not used the logistic regression will be able to predict a probability of usage.

SUMs for three stone fires were placed in a SUM holder and then placed under one of the stones in the three stone fire. The SUMs for Envirofits were attached using duct tape and wire and placed at the base of the stove behind the intake location for the firewood.

*Visual Observations of Use*

Each time any part of data collection team visited a household he or she visually observed which stoves were in use (whether the stove was “on” or “off” along with the date and timestamp recorded digitally via handheld device). Enumerators visited a house numerous times during a “measurement week,” when we also enumerated a survey and weighed wood for the kitchen performance test. Another enumerator visited once every 4-6 weeks to download data and reset the SUMs device.

*Generating an Algorithm*

Our technique requires continuous SUMs temperature data for a given stove, and recorded instances of whether that particular stove is seen in use or not. We matched visual observations of stove use to SUMs temperature data by time and date stamps. The core of our method is a logistic regression using the lags and leads of the SUMs temperature data to predict visual observations of stove usage. We tested ten specifications of differing combinations of current, lagged, and leading temperature readings (Simons et al. 2014).

In order to determine which of the models was most appropriate we test the ten specifications with the Akaike information criterion (AIC) (Akaike 1981). The AIC trades off goodness of fit of the model with the complexity of the model to guard against over fitting.

The preferred specification included the temperature reading closest to the time of the visual observation, the readings 60 and 30 minutes prior, and 60 and 30 minutes after the visual observation of use, and a control for hour of the day. This regression specification correctly predicted 89.3% of three stone fire observations and 93.8% of Envirofit observations of stove usage. We then compared our algorithm to other previously published algorithms (Ruiz-Mercado, Canuz, and Smith 2012; Mukhopadhyay et al. 2012). Those algorithms focused on defining “discrete” cooking events based on rapid temperature slope increases, elevated stove temperatures, and then followed by a cooling off period. We applied those algorithms to the temperature data we collected and found our logistic regression correctly classified more observations, with a higher pseudo R-squared, than any other algorithm for both three stone fires and the Envirofits.

*Kitchen Performance Test Protocol*

The kitchen performance test weights the woodpile in a kitchen on sequential days to quantify the amount of wood used in a given 24-hour period (for additional details see: <http://cleancookstoves.org/technology-and-fuels/testing/protocols.html>). The KPT is the protocol used to estimate fuel savings, a primary component of calculating carbon credits for a stove project (The Gold Standard Foundation 2013). To minimize variance, the standard recommendation is that the KPT testing period should be at least three days, avoiding weekends and holidays (Bailis, Smith, and Edwards 2007b).

On the initial visit of the KPT week, the data collection team asked the household cook to describe what fuels they would use in the next 24-hour period. The data collection team asked the household to stack the wood they expected to use in a pile and only use wood from that pile over the next 24 hours. To ensure that the household did not run out of fuel, we asked the household to add a few extra pieces to the pile before we weighed the pile. In approximately 24 hours, the data collection team returned and weighed the remaining fuel. This process was repeated at approximately the same time each day of the KPT week (Monday – Thursday).

We measured mean 24-hour concentrations of PM2.5 by installing calibrated UCB-PATS PM monitors in the study participants' homes during the same 72 hours of the kitchen performance test. We followed best practices as outlined by the Berkeley Air Monitoring Group (see: http://berkeleyair.com/services/ucb-particle-and-temperature- sensor-ucb-pats/) and measured three consecutive days of mean 24-hour PM2.5 concentrations in the kitchen. We averaged data from the UCB- PATS PM monitors into 24-hour average PM2.5 readings in μg/m^3^.

*Data used for Hawthorne effect analysis*

Comparing stove usage calculated from the temperature data collected by the SUMs in the week while KPT measurement teams are present versus stove usage in the week before and after the measurement week provides our test of a Hawthorne effect (Simons et al. in press, 2016). We also use data from the baseline KPT period to have a baseline understanding of how much fuelwood is consumed and the indoor particulate matter concentrations when households were operating (generally with two, three-stone fires), prior to the introduction of any fuel-efficient stoves. Stata data files and do files are included in the data in brief article.

**Acknowledgements** This data collection was funded by the United States Agency for International Development under Translating Research into Action, Cooperative Agreement No. GHS-A-00-09-00015-00. The recipient of the grant was Impact Carbon who co-funded and managed the project. Juliet Kyaesimira and Stephen Harrell expertly oversaw field operations and Amy Gu provided excellent research support. Further, we thank Impact Carbon partners Matt Evans, Evan Haigler, Jimmy Tran, Caitlyn Toombs, and Johanna Young; U.C. Berkeley Household Energy, Climate, and Health Research Group partners including Kirk Smith, Ilse Ruiz-Mercado, and Ajay Pillarisetti; Berkeley Air partners including Dana Charron, David Pennise, Michael Johnson, and Erin Milner. Data collection was carried out by the Center for Integrated Research and Community Development (CIRCODU), and the project’s success relied on expert oversight by CIRCODU's Director General Joseph Ndemere Arineitwe and field supervisors Moreen Akankunda, Innocent Byaruhanga, Fred Isabirye, Noah Kirabo, and Michael Mukembo. We thank the Atkinson Center for a Sustainable Future at Cornell University, the Institute for the Social Sciences at Cornell University and the Cornell Population Center for additional funding of related expenses. The findings of this study are the sole responsibility of the authors, and do not necessarily reflect the views of their respective institutions, nor USAID or the United States Government.

**References** *[please include all references relevant to the data described here; references are not limited]*

Akaike, Hirotugu. 1981. “Likelihood of a Model and Information Criteria.” *Journal of Econometrics* 16: 3–14.

Bailis, Rob, Kirk R. Smith, and Rufus Edwards. 2007a. “Kitchen Performance Test (KPT).” Univerisity of California, Berkeley, CA.

Bailis, Rob, Kirk R Smith, and Rufus D. Edwards. 2007b. “Kitchen Performance Test (KPT).”

Beltramo, Theresa, Garrick Blalock, David I. Levine, and Andrew M. Simons. 2015. “Does Peer Use Influence Adoption of Efficient Cookstoves? Evidence From a Randomized Controlled Trial in Uganda.” *Journal of Health Communication* 20 (S1): 55–66. doi:10.1080/10810730.2014.994244.

Beltramo, Theresa, David I. Levine, Garrick Blalock, and Andrew M. Simons. 2015. “The Effect of Marketing Messages and Payment over Time on Willingness to Pay for Fuel-Efficient Cookstoves.” *Journal of Economic Behavior & Organization*. doi:10.1016/j.jebo.2015.04.025.

Chowdhury, Zohir, Rufus Edwards, Michael Johnson, Kyra Naumoff Shields, Tracy Allen, Eduardo Canuz, and Kirk R Smith. 2007. “An Inexpensive Light-Scattering Particle Monitor: Field Validation.” *Journal of Environmental Monitoring* 9 (10): 1099–1106.

Harrell, Stephen, Theresa Beltramo, Garrick Blalock, Juliet Kyayesimira, David I. Levine, and Andrew M. Simons. 2016. “What Is a Meal?: Comparing Methods of Auditing Carbon Offset Compliance for Fuel Efficient Cookstoves.” *Ecological Economics* 128: 8–16.

Levine, David I, Theresa Beltramo, Garrick Blalock, Carolyn Cotterman, and Andrew M. Simons. 2016. “What Impedes Efficient Adoption of Products? Evidence from Randomized Sales Offers for Fuel-Efficient Cookstoves in Uganda.” Berkeley, CA. http://escholarship.org/uc/item/2cb9g10z.

Mukhopadhyay, Rupak, Sankar Sambandam, Ajay Pillarisetti, Darby Jack, Krishnendu Mukhopadhyay, Kalpana Balakrishnan, Mayur Vaswani, et al. 2012. “Cooking Practices, Air Quality, and the Acceptability of Advanced Cookstoves in Haryana, India: An Exploratory Study to Inform Large-Scale Interventions.” *Global Health Action* 5: 19016–19016.

Pillarisetti, Ajay, Mayur Vaswani, Darby Jack, Kalpana Balakrishnan, Michael N Bates, Narendra K Arora, and Kirk R Smith. 2014. “Patterns of Stove Usage after Introduction of an Advanced Cookstove: The Long-Term Application of Household Sensors.” *Environmental Science & Technology* 48 (24): 14525–14533.

Ruiz-Mercado, Ilse, Eduardo Canuz, and Kirk R. Smith. 2012. “Temperature Dataloggers as Stove Use Monitors (SUMs): Field Methods and Signal Analysis.” *Biomass and Bioenergy* 47: 459–68. doi:10.1016/j.biombioe.2012.09.003.

Ruiz-Mercado, Ilse, Eduardo Canuz, Joan L. Walker, and Kirk R. Smith. 2013. “Quantitative Metrics of Stove Adoption Using Stove Use Monitors (SUMs).” *Biomass and Bioenergy* 57 (October): 136–48.

Ruiz-Mercado, Ilse, Nick L Lam, Eduardo Canuz, Gilberto Davila, and Kirk R Smith. 2008. “Low-Cost Temperature Loggers as Stove Use Monitors (SUMs).” *Boiling Point* 55: 16–18.

Simons, Andrew M., Theresa Beltramo, Garrick Blalock, and David I. Levine. in press. “Using Unobtrusive Sensors to Measure and Minimize Hawthorne Effects: Evidence from Cookstoves.” *Journal of Environmental Economics and Management*.

———. 2014. “Comparing Methods for Signal Analysis of Temperature Readings from Stove Use Monitors.” *Biomass and Bioenergy* 70: 476–88.

———. 2016. “Using Unobtrusive Sensors to Measure and Minimize Hawthorne Effects: Evidence from Cookstoves.” *Dyson School of Applied Economics and Management Working Paper*.

The Gold Standard Foundation. 2013. “The Gold Standard: Simplified Methodology for Efficient Cookstoves.” Geneva-Cointrin, Switzerland.

1. The SUMs used for our project, iButtons™ manufactured by Maxim Integrated Products, Inc., are small stainless steel temperature sensors about the size of a small coin and the thickness of a watch battery which can be affixed to any stove type. Our SUMs record temperatures with an accuracy of +/- 1.3 degrees C up to 85°C. For additional details see the product description website at: <http://berkeleyair.com/services/stove-use-monitoring-system-sums/> The SUMs cost approximately USD$16 each. They recorded temperature data every 30 minutes for six weeks in a household before needing minimal servicing from a technician to download the data. After the data download they can be reset and re-used. [↑](#footnote-ref-1)
2. We used UCB Particle and Temperature Sensors (UCB-PATS) to measure air pollution particles that were 2.5 micrometers in diameter or smaller (PM2.5). Due to their small size these particles can get deep into the lungs and are the single best indicator of risk for many respiratory related diseases (Chowdhury et al. 2007). [↑](#footnote-ref-2)
3. Overnight, while most participants report sleeping, SUMs record the residual heat absorbed in the large stones of the three stone fires and/or from coals banked overnight. Therefore our algorithm overestimates overnight cooking of three stone fires. We adjust for this in the subsequent analysis. For further discussion and a description of the technical adjustment see Harrell et al. (2016). [↑](#footnote-ref-3)
