# Supplementary material for: Sensor data to measure Hawthorne effects in cookstove evaluation
Source: Data Brief. 2018 Apr 11;18:1334–9. doi: 10.1016/j.dib.2018.04.021 (PMC5997008; doi:10.1016/j.dib.2018.04.021)
Supplement: Supplementary file 2 — Supplementary material [file mmc2.zip › Data_in_Brief/Hawthorne_Effects_Data_Do_Files_27Feb17/Readme_File_Code_and_Data_Hawthorne_Effect.docx]

Readme file for data and code for

*Using Unobtrusive Sensors to Measure and Minimize Hawthorne Effects: Evidence from Cookstoves*

by Simons, Beltramo, Blalock, Levine

**Overview:** The do file RCT3_SUMs_Hawthorne_Replication_Table.do makes the main Hawthorne effect table using the endline data. The do file RCT3_SUMs_Hawthorne_PM_Replication.do runs regressions on wood usage, PM2.5, and total stove use prior to introduction of Envirofits. The do file RCT3_SUMs_Hawthorne_Replication_Attrition_Checks.do runs the regressions that check if SUMs attrition is non-random. The do files only creates partial .tex files and then the end user must manually make some edits/additions to the .tex files to make them more visually appealing. In order to use this do file please follow the following folder structure:

**Data Folder Structure:** create a higher-level folder with the following sub folders:

clean_data

do_files

logs

raw_data

tables

Then place the do files into the folder called do_files. The user must define his or her own path before running the do file (delete the existing path under library definitions and insert your computer path). When running the do file the other folder locations are specified with global variables at the beginning of the do file so once the user has inserted his or her path at the beginning of the do file, then the do file will make global variables relative to that path for the five folders outlined above.

**Data:** place the four data files called RCT3_SUMSs_Hawthorne_Replication.dta, RCT3_SUMs_Hawthorne_PM_Replication.dta, HH_in_Hawthorne_Regression.dta, and RCT3_SUMs_Hawthorne_Attrition_Checks_Replication.dta into the raw_data folder. The replication of all tables and figures in the paper will work just by running the do file once as long as the two data files are inserted into the correct folders and the data folder structure as laid out above is followed. Note, some tables in the final paper are calculated simply multiplying certain numbers; these are not included in the do files.

**Logs:** every time the do file is run a new log file with the time and date stamp will be written and saved in the log folder

**Tables:** the do file will save .tex files in the tables folder, those are the raw .tex files used to generate the tables for the final paper, however these files require some editing to be more visually pleasing.
